# A fast jaw‐tracking model for VMAT and IMRT Monte Carlo simulations

**DOI:** 10.1002/acm2.12343

**Published:** 2018-05-09

**Authors:** Reid Townson, Hilary Egglestone, Sergei Zavgorodni

**Affiliations:** ^1^ Measurement Science and Standards National Research Council Canada Ottawa ON Canada; ^2^ Department of Physics and Astronomy University of Victoria Victoria BC Canada; ^3^ Department of Medical Physics BC Cancer Agency Vancouver Island Centre Victoria BC Canada

**Keywords:** dose calculation, Monte Carlo, radiotherapy, VMAT

## Abstract

Modern radiotherapy techniques involve routine use of volumetric arc therapy (VMAT) and intensity modulated radiotherapy (IMRT) with jaw‐tracking – dynamic motion of the secondary collimators (jaws) in tandem with multi‐leaf collimators (MLCs). These modalities require accurate dose calculations for the purposes of treatment planning and dose verification. Monte Carlo (MC) methods for radiotherapy dose calculation are widely accepted as capable of achieving high accuracy. This paper presents an efficiency‐enhancement method for secondary collimator modeling, presented in the context of a tool for MC‐based dose second checks. The model constitutes an accuracy trade‐off in the source model for the sake of efficiency enhancement, but maintains the advantages of MC transport in patient heterogeneities. The secondary collimator model is called Flat‐Absorbing‐Jaw‐Tracking (FAJT). Transmission through and scatter from the secondary collimators is neglected, and jaws are modeled as perfectly absorbing planes. To couple the motion of secondary collimators with MLCs for jaw‐tracking, the FAJT model was built into the VCU‐MLC model. Gamma‐index analysis of the dose distributions from FAJT against the full BEAMnrc MC simulations showed over 99% pass rate for a range of open fields, two clinical IMRT, and one VMAT treatment plan, for 2%/2 mm criteria above 10%. Using FAJT, the simulation speed of the secondary collimators for open fields increased by a factor of 237, 1489, and 1395 for 4 × 4, 10 × 10, and 30 × 30 cm^2^, respectively. In general, clinically oriented simulation times are reduced from “hours” to “minutes” on identical hardware. Results for nine representative clinical cases (seven with jaw‐tracking) are presented. The average 2%/2 mm *γ*‐test success rate above the 80% isodose was 96.8% when tested against the EPIDose electronic portal image‐based dose reconstruction method and 97.3% against the Eclipse analytical anisotropic algorithm.

## 1 INTRODUCTION

Dose calculations in radiation therapy have been performed by algorithms of varying complexity and accuracy with calculations based on Monte Carlo (MC) methods being arguably the most accurate in complex geometries and heterogeneous media encountered in clinical dosimetry and treatment planning.^1^, ^2^, ^3^, ^4^, ^5^, ^6^, ^7^, ^8^ For this reason, MC techniques are expected to play a substantial role in radiotherapy treatment dose calculations and verification in the foreseeable future. However, complete MC simulations of the linear accelerator head and patient geometry can be computationally expensive and require calculation times prohibitively long for use during the treatment planning and dose verification stages. Modern generations of fast dose calculation codes employ advanced variance reduction techniques to dramatically improve dose calculation efficiency in the phantom – this leaves treatment head modeling as the computational bottleneck. The problem is partially resolved using phase‐space sources, which store particle fluence after simulation of treatment plan‐independent components. Additionally, some linac manufacturers provide phase‐space models to users rather than geometrical specifications. As such, many strategies for fast source models involve the use of phase‐space files.^9^, ^10^, ^11^, ^12^, ^13^

When modeling the treatment head, many of the primary particles are absorbed in the secondary collimators and do not contribute to the dose in the volume of interest. This is the usual bottleneck on simulation time, particularly when combined with a fast MLC model^14^, ^15^ and a dose calculation code such as VMC++,^16^ DPM^17^, and gDPM.^18^ A number of fast MC codes have been summarized recently.^19^ Analytical (or semi‐analytical) source models can achieve high efficiency, particularly on GPU devices.^13^, ^20^ However, many users are dependent on phase‐space sources and do not have secure access to GPU resources – this is the context for the present article.

A previous study^21^ investigated the effects of simplified particle transport through the secondary collimators on calculation efficiency. In particular, the authors presented a planar, completely absorbing collimator model positioned at the vertical midpoint of each jaw. This model ignored collimator transmission and scatter, and achieved a 274‐fold gain in overall efficiency and good agreement with a MC benchmark for a 6 MV 10 × 10 cm^2^ field. However, clinical dose distributions had relatively low agreement with a benchmark, dropping from gamma‐index test results (1%/1 mm) of 98%–97% for more rigorous models to 65%–68% for the flat‐absorbing model. These results were discouraging and no following investigations of similar models have been reported in the literature.

In this paper, we present and evaluate a similar secondary collimator model, but combined with a more sophisticated MLC model. The implementation includes dynamic motion of secondary collimators in jaw‐tracking mode and dynamic MLC modeling for VMAT and IMRT dose calculations. The jaws are simulated as perfectly absorbing planes positioned at the top surface of each secondary collimator. This jaw model (called Flat‐Absorbing‐Jaw‐Tracking, or FAJT) was integrated into the VCU‐MLC model^14^, ^15^ to enable fast simulation of secondary collimator motions coupled with MLC motion in jaw‐tracking. While full particle transport models with jaw‐tracking have been previously reported,^22^, ^23^, ^24^ the FAJT model achieves higher efficiency while maintaining sufficient accuracy for a range of clinical applications, particularly treatment planning dose verification.

## 2 METHODS

**Figure 1:**
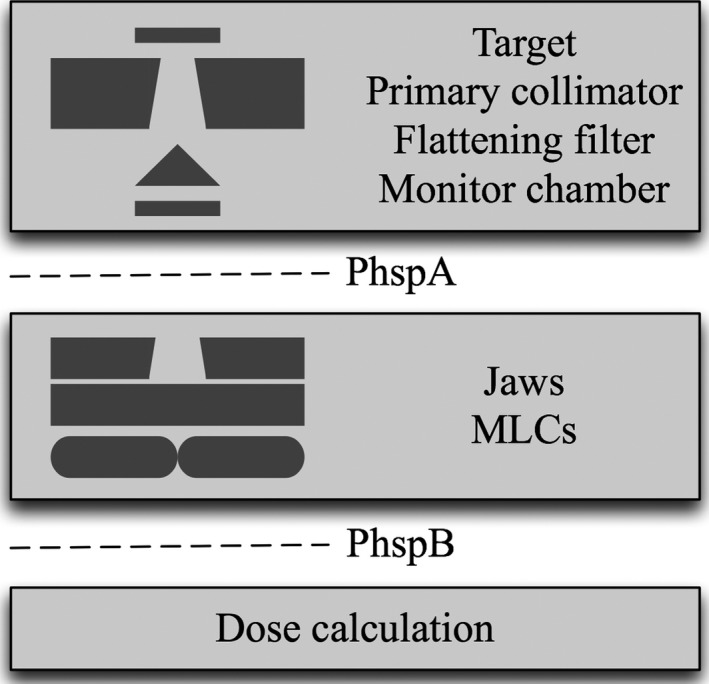
An illustration of the linac Monte Carlo model and intermediate phase‐spaces.

**Figure 2:**
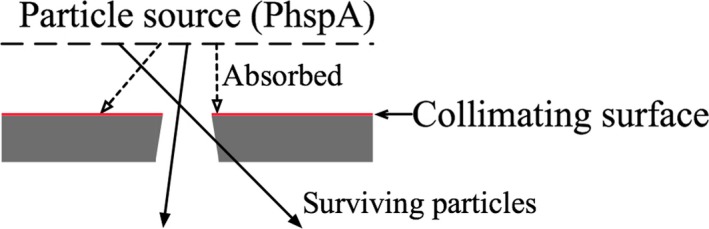
An illustration of the FAJT collimation process. Only particles that strike the top surface of the collimator are absorbed (open arrows). Otherwise, the particles are projected to the next collimator without scattering (closed arrows).

The FAJT method is a fast alternative to performing MC simulation of photon and electron transport through secondary collimators. It presents the jaws as perfectly absorbing planes positioned at the top (closest to the target) surface of each of the secondary collimators. An essential component of achieving high efficiency with this model is azimuthal particle redistribution^25^, ^26^ (APR). APR is a variance reduction technique usually used to suppress latent variance^17^ from nonanalytic (phase‐space) sources. In this work, we will refer to such an incident phase‐space source as PhspA, as shown in Fig. 1. Each particle from the source is recycled a number of times and azimuthally redistributed. After performing APR, particles are ray‐traced to the top of the secondary collimators to determine whether or not they pass through the collimator opening. Figure 2 illustrates an example of absorbed and allowed particles. Those particles that pass within the collimator opening are kept for further simulation (eventually written to an intermediate phase‐space that will be used for dose calculation, called PhspB).

For most linac source models, the source particles are largely diverging from a small spot, so the choice of collimation surface at the top, middle, or bottom of each jaw has little impact on results. The top surface was chosen to eliminate both diverging and “downward” aimed particles most effectively (imagine a parallel beam as an extreme case).

### 2.A The jaw model for open fields

Open fields are now practically obsolete in clinical practice, but they are important for model benchmarking and evaluating effects of potential approximations. Therefore, a simplified version of the FAJT model has been implemented for open field modeling.

The basic input parameter required by the dose calculation engine is number of particles to simulate ($N^{\mathrm{requested}}$); this number is determined from statistical uncertainty estimations (not shown here). To avoid restarting the phase‐space in the dose calculations, the phase‐space PhspB scored below the secondary collimator is generated to contain exactly the number of particles that will be simulated ($N^{\mathrm{requested}}$). The number of particles read from the input phase‐space PhspA $N^{\mathrm{read}}$ is determined on‐the‐fly based on the number of rejected particles, in order to achieve $N^{\mathrm{requested}}$. Recycling of the PhspA combined with APR is used to avoid very large input phase‐space files, which allows for the data to be stored in RAM prior to the simulation for high‐speed access. Additionally, particles outside the radius of the maximum collimator opening can be immediately discarded, while those within the field are recycled. The number of times to recycle each particle from PhspA is set to a fixed number, $N^{\mathrm{recycle}}$, which is chosen with the aim of being large enough to avoid reading PhspA more than once, and small enough to avoid latent variance. The first particle to be read from the PhspA is chosen at random to ensure the independence of parallelized calculations, and subsequent particles are read sequentially from the file.

### 2.B The jaw‐tracking model for VMAT and IMRT

The flat‐absorbing jaws were integrated into the VCU‐MLC model^14^, ^15^ that was designed for fast simulation of radiation transport through moving collimator leaves. The VCU‐MLC software uses MLC control points from the treatment plan to specify the positions of each leaf during radiation delivery. Each control point is associated with fractional delivered monitor units (MUs). In the original implementation of VCU‐MLC without an integrated jaw model, the particle source for MLC simulation was a PhspB from a BEAMnrc^27^ simulation of the secondary collimators (scored just above the top of the MLCs). As each particle is read from the phase‐space, positions of the MLCs for the particle to be transported through are determined by randomly sampling the fractional MU delivered. The particle is then transported through the MLCs using exact MLC geometry but an approximate transport model.

In jaw‐tracking mode, every control point also contains positions of each jaw. To model jaw‐tracking, the VCU‐MLC code was modified to include modeling secondary collimators as flat‐absorbing jaws. In this case, particles originate from PhspA particle source stored in RAM, and APR is applied to each particle as described in the previous section. The fractional MUs are randomly sampled for each particle. This determines both the jaw positions and the corresponding MLC positions. The jaw positions are then used to determine if the particle gets absorbed or survives in the flat‐absorbing jaw model. The particles which survive through the jaws are projected past the jaws and included for transport through the MLCs using the VCU‐MLC model. This enables the synchronization of MLC and secondary collimator motions. Finally, particles transported through the collimators are scored to a new phase‐space just below the MLC to be further transported into a phantom for the dose calculations.

### 2.C Absolute dose calculation

All MC simulations were performed using our in‐house Monte Carlo software framework.^28^, ^29^ Within the framework, transport through a phantom is performed using either the DOSXYZnrc^30^ or VMC++^16^ dose calculation software. It has been shown that the dose calculations from these codes are in excellent agreement.^31^ However, implementation of the FAJT model is independent of the dose calculation code. We have chosen to highlight VMC++ in this text because its fast simulation speeds make it particularly well‐suited to the task.

When the MC dose calculation is complete, the dose distribution $D_{o}$ in relative dose units of Gy per initial electron has to be converted to dose in units of Gy to enable comparison with other dose calculation methods or experimental measurements. To convert from the initial dose $D_{o}$ to units of Gy for a given number of monitor units, MU, the following approach was used. Assume the calibration was performed in source‐to‐axis distance (SAD) conditions (a 10 × 10 cm^2^ field, 90 cm source‐to‐surface distance (SSD) in water, reference depth $d_{\mathrm{ref}}=10\mathrm{cm}$). The tissue maximum ratio (TMR) along with calibration dose $D^{\mathrm{cal}}$ (Gy) at $d^{\max}$ can be used to reflect the measurement, $D^{\mathrm{cal}}$ at depth $d_{\mathrm{ref}}$. Then the dose $D^{\prime }$ in units of Gy is

(1) $$D^{\prime }=D_{o}\frac{\mathrm{TMR}\left(d_{\mathrm{ref}},10\times 10\right)\cdot D^{\mathrm{cal}}\left(d^{\max},10\times 10\right)}{D^{\mathrm{MC}}\left(d_{\mathrm{ref}},10\times 10\right)}\cdot \mathrm{MU}\cdot S_{b}$$

where $S_{b}$ accounts for backscatter radiation from the secondary collimators into the monitor chamber.^32^ The backscatter correction is necessary since MC models generally do not account for the experimental effect of backscatter into the monitor chamber, a mechanism that impacts the dose delivered and depends on collimator positions.

In full simulation of the treatment head $S_{b}$ can be obtained by recording the dose in the monitor chamber separately for the forward (toward the phantom) or backward moving particles. However, without scatter from the secondary collimators modeled in FAJT, it was instead necessary to use a measurement‐based $S_{b}$ look‐up table, determined in previous work.^33^

To account for dynamic collimator motion, a value of $S_{b}$ was determined separately for each control point in an IMRT plan, using a look‐up table and the secondary collimator positions. Each $S_{b}$ factor was then assigned a weighting factor according to the fractional MU associated with the given control point relative to the total MU in the field. Finally, using a weighted average over the control points, $S_{b}$ was calculated for each field in IMRT jaw‐tracking plans. VMAT plans were treated differently – gantry motion in our VMAT MC model^28^ was simulated by splitting each arc in sub‐fields corresponding to pairs of control points. VMAT plans use $S_{b}$ values calculated and applied separately to each sub‐field.

### 2.D Simulation hardware and parallel processing

The computations in this work utilized three compute nodes, each with four AMD Opteron 2.1 GHz 16‐core processors, 192 GB DDR3 RAM, and 7200 RPM SATA hard drives. One of the nodes itself acts as a front‐end job submission host, and distributes jobs to the other nodes using the Condor batching system. For IMRT treatment plans, each treatment field (static gantry angle) is split into $N^{\mathrm{split}}$ identical sub‐fields to enable parallelization over a greater number of CPU cores. Upon completion of all sub‐field calculations, the dose distributions are cumulated into a total dose distribution for the field. Then the fields are converted to absolute dose and cumulated. This process is similar for VMAT plans, but the sub‐fields are created for each pair of control points (fractional MU steps) and are not identical. The last CPU core to finish dose calculations performs summation of the results from all parallel cores. This portion of the post processing tends to be more time consuming for VMAT plans, where the simulation is divided into a large number of independent parallel simulations to discretely model dynamic gantry rotation.

### 2.E Validation and performance tests

The validation results presented in this paper include a range of clinical VMAT and IMRT cases, as well as a number of open fields. FAJT was compared with the gold standard for MC linac simulation, BEAMnrc, as well as an EPID‐based dose reconstruction technique and the analytical anisotropic algorithm (AAA) in the Eclipse treatment planning system. In all MC simulations, the number of particles simulated was estimated such that <1% statistical uncertainty (of the local voxel dose) was achieved in the majority of voxels containing >10% of the maximum dose. The comparison simulations were performed on the same hardware, utilizing the same number of cores to enable a fair competition.

Open fields in water were calculated to evaluate the accuracy of the FAJT secondary collimator modeling, compared to full MC simulation with BEAMnrc. A virtual water phantom was used, positioned at an 80, 90, and 100 cm source‐to‐surface distance (SSD) and comprised of 82 × 82 × 82 voxels with 5‐mm voxel resolution. The open field sizes 4 × 4, 10 × 10 and 30 × 30 cm^2^ were simulated with the phantom. The accelerator modeled was a 6 MV Varian TrueBeam, using a previously validated phase‐space source (PhspA).

As the benchmark for comparison, BEAMnrc 2008 was used with components modeling the monitor chamber, the MCTWIST module for APR,^24^ and secondary collimators. The same PhspA was used as an input source. The energy cutoffs ECUT and PCUT were 0.7 and 0.01 MeV, respectively. All BEAMnrc transport parameters are shown in Table 1. Automatic recycling was enabled in BEAMnrc, which means that the number of recyclings was calculated as $N^{\mathrm{requested}}/N^{\mathrm{phsp}}$, rounded up. When using the FAJT method, the number of recyclings (with APR) was set to 20.

**Table 1:** The BEAMnrc transport parameters. Any parameters not shown were set to default values

BEAMnrc parameter	Value
Global ECUT	0.7
Global PCUT	0.01
Global SMAX	5
ESTEPE	0.25
XIMAX	0.5
Boundary crossing algorithm	PRESTA‐I
Skin depth for BCA	0
Electron‐step algorithm	PRESTA‐II
Spin effects	On
Brems angular sampling	Simple
Brems cross‐sections	BH
Bound Compton scattering	Off
Pair angular sampling	Simple
Photoelectron angular sampling	Off
Rayleigh scattering	Off
Atomic relaxations	Off
Electron impact ionization	Off

Three clinical patient treatment plans are presented to demonstrate the functionality of our implementation of the FAJT model and compare with BEAMnrc calculations: an IMRT brain treatment verification plan calculated in a homogeneous water cylinder (IMRT 1), an IMRT esophagus case (IMRT 2), and a VMAT lung case (VMAT 1) (Table 2). Patient phantoms for MC simulation were generated from CT scans into a suitable format with down‐sampled resolution. All of the virtual patient phantoms were created with 5 × 5 × 5 mm^3^ voxel size (except where otherwise specified).

**Table 2:** 3D dose analysis with FAJT as the evaluation dose and BEAMnrc as the reference. Comparisons were performed only in voxels containing >10% of the dose in the reference. The IMRT brain case plan was calculated in a homogeneous water cylinder, while the other IMRT and VMAT cases used heterogeneous patient phantoms

Plan	*γ* (%) 2%/2 mm	*χ* (%) 2%/2 mm	RMSD (%)
4 × 4 SSD = 80 cm	99.9	100	0.6
4 × 4 SSD = 90 cm	100	99.9	0.5
4 × 4 SSD = 100 cm	99.9	100	0.6
10 × 10 SSD = 80 cm	100	100	0.4
10 × 10 SSD = 90 cm	100	98.9	0.4
10 × 10 SSD = 100 cm	100	100	0.5
30 × 30 SSD = 80 cm	99.9	100	0.4
30 × 30 SSD = 90 cm	100	98.2	0.5
30 × 30 SSD = 100 cm	100	100	0.5
IMRT 1 Brain (cylinder)	99.6	99.4	0.6
IMRT 2 Esophagus	99.9	99.5	0.6
VMAT 1 Lung	100	99.8	0.7

To test the model against measured data, including the jaw‐tracking implementation, comparisons were made against the portal image‐based 3D dose reconstruction method “EPIDose”.^34^ This software has been in clinical use for over 10 yr and has been used as a primary QA tool for thousands of IMRT and VMAT plans. For these evaluations, clinical patient plans were converted into verification plans calculated in a cylindrical water phantom of 20.4 cm diameter with a voxel size of 2.5 × 2.5 × 2.5 mm^3^. Experimental measurements using an electronic portal imaging device (EPID) are used in the dose reconstruction. FAJT was also compared with AAA dose calculations. The AAA algorithm in our clinic has been thoroughly configured and provides accurate results in homogeneous phantoms, such as the water cylinder used in these tests.

Nine clinical plans (three IMRT and six VMAT, seven with jaw‐tracking) were selected to provide a diverse and representative range of treatment sites and include both large and small planning target volumes (PTVs). The plans with jaw‐tracking enabled were not compared with BEAMnrc because this capability has not yet been implemented in our software framework. Comparisons were made using root mean square deviation (RMSD), 3D *γ*‐index, and 3D *χ*‐index tests.^35^, ^36^ Both *γ*‐ and *χ*‐index test results are presented for the reader's consideration, due to the susceptibility of *γ*‐index to bias from statistical fluctuations in dose distributions. The *χ*‐index is a similar metric, but a gradient weighted dose difference that may be more robust. Both *γ*‐ and *χ*‐index test criteria were 2%/2 mm using only voxels above 10% of the maximum dose (unless otherwise specified), with FAJT as the evaluation dose and AAA or EPIDose as the reference dose.

## 3 RESULTS

**Figure 3:**
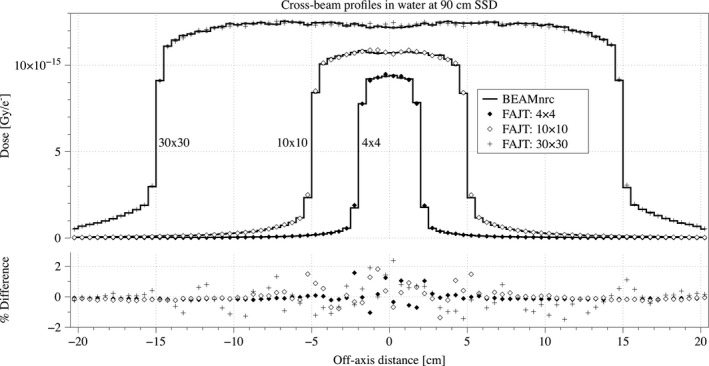
Cross‐beam profiles at 10 cm depth and SSD = 90 cm are shown in units of Gy/e‐ for the FAJT method (dots) and the benchmark, BEAMnrc (lines) derived from the same initial phase‐space. Statistical uncertainties are shown only for the BEAMnrc curves. The percentage differences are also shown, relative to the maximum benchmark dose in the curve.

**Figure 4:**
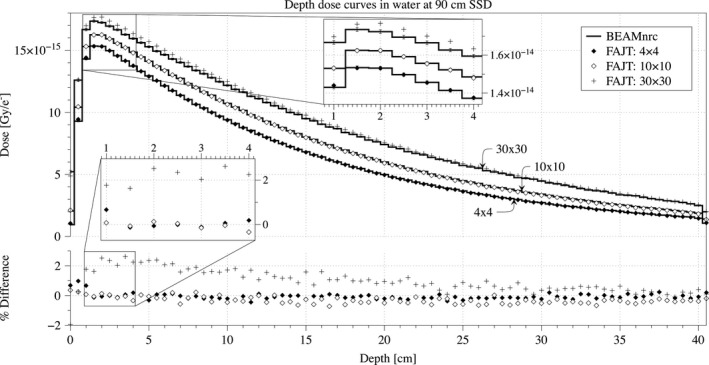
Depth dose curves at SSD = 90 cm are shown in units of Gy/e‐ for the FAJT method (dots) and the benchmark, BEAMnrc (lines) derived from the same initial phase‐space. Statistical uncertainties are shown only for the BEAMnrc curves. The percentage differences are also shown, relative to the maximum benchmark dose in the curve.

**Figure 5:**
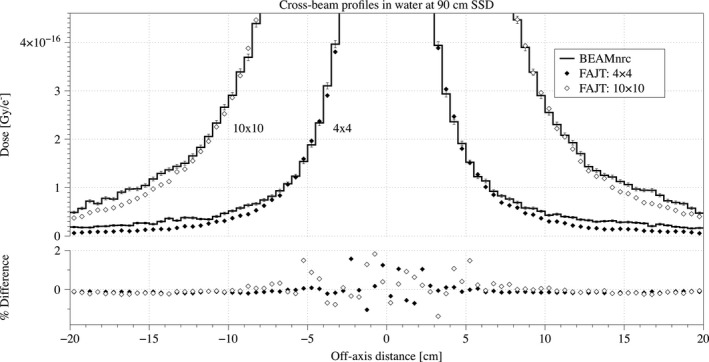
The same as Fig. 1, but zoomed to highlight out‐of‐field systematic error. Notice that the magnitude of the error out‐of‐field is on the order of 0.1% of the maximum dose.

Cross‐beam profiles and depth dose curves with SSD = 90 cm are shown in Figs. 3 and 4, respectively. The overall agreement of open fields is good and quantified using RMSD, *γ*‐and *χ*‐index tests in Table 2. Even in out‐of‐field region where FAJT is expected to underestimate the dose compared to BEAMnrc, we only see a very small difference amounting to ~0.1% of the central axis dose maximum, as shown in Fig. 5. Analysis of FAJT produced dose distributions compared to BEAMnrc calculations showed that, for open fields as well as the three clinical cases, *γ*‐index test agreement was very good (>99% for 2%/2 mm criteria above the 10% isodose).

In the clinical verification plans that were compared with EPIDose reconstruction and AAA calculations, *γ*‐index pass rates in regions above the 40% isodose consistently exceeded 95% (Table 3). Lower pass rates were seen in the regions with doses in the 20%–40% range. However, pass statistics for FAJT MC calculations are very consistent with those by AAA and the dose distributions are in better agreement. The reduced agreement with EPIDose is attributed primarily to imperfection of the portal image‐based reconstruction algorithm rather than FAJT MC model, as discussed in the following section.

**Table 3:** *γ*/*χ* pass rates for the evaluation/reference pairs: FAJT/EPIdose, AAA/EPIDose, and FAJT/AAA. The *γ*‐/*χ*‐index criterion was 2%/2 mm. The equivalent diameter of the PTV volume is provided

Plan	Isodose range (%)	FAJT vs EPIDose	AAA vs EPIDose *γ*/*χ* (%)	FAJT vs AAA
JT‐VMAT	>80	90.8/92.3	94.8/96.7	97.5/98.8
Intra‐cranial	40–80	99.9/100	99.8/100	99.5/100.0
Eq. D: 2.9 cm	20–40	98.7/99.1	98.9/99.2	100.0/100.0
JT‐VMAT	>80	99.6/99.8	99.8/99.8	99.9/100.0
Scalp	40–80	97.4/97.5	97.7/97.9	99.9/100.0
Eq. D: 8.7 cm	20–40	91.4/91.1	91.6/91.6	99.9/100.0
JT‐IMRT	>80	99.4/99.3	99.6/99.8	99.6/98.8
L Brain (CNS)	40–80	97.9/93.6	98.1/95.8	100.0/99.9
Eq. D: 8.9 cm	20–40	88.6/89.0	90.2/90.5	99.8/100.0
JT‐VMAT	>80	99.8/99.9	99.3/99.4	99.8/100.0
L Lung	40–80	98.1/98.2	98.3/98.4	100.0/100.0
Eq. D: 9.4 cm	20–40	95.5/96.3	96.0/96.3	99.9/100.0
IMRT	>80	96.8/97.4	96.4/97.1	99.0/100.0
Gastric Bed	40–80	96.6/95.0	97.2/95.3	99.7/99.9
Eq. D: 10.6 cm	20–40	89.2/89.4	90.9/91.0	99.7/99.9
JT‐VMAT	>80	99.6/99.9	99.5/99.9	99.3/100.0
Larynx H&N	40–80	99.6/99.7	99.8/99.8	99.9/100.0
Eq. D: 5.4 cm, 8.3 cm	20–40	94.2/95.0	96.0/96.5	100.0/100.0
JT‐VMAT	>80	98.1/98.6	98.5/98.8	99.5/100.0
Esophagus	40–80	98.4/98.8	99.5/99.6	100.0/100.0
Eq. D: 16.4 cm	20–40	93.8/96.2	90.6/92.3	98.0/100.0
IMRT	>80	97.3/97.3	98.9/98.9	100.0/99.9
Vagina and nodes	40–80	96.2/96.6	96.3/96.7	100.0/100.0
Eq. D: 7.5 cm, 11.1 cm	20–40	91.8/93.7	90.6/92.1	99.2/100.0
JT‐VMAT	>80	96.8/97.8	97.1/98.2	99.8/100.0
Anus and nodes	40–80	95.2/96.4	95.4/96.4	99.9/100.0
Eq. D: 11.1 cm, 9.2 cm	20–40	93.7/96.2	91.9/94.5	97.1/100.0

**Figure 6:**
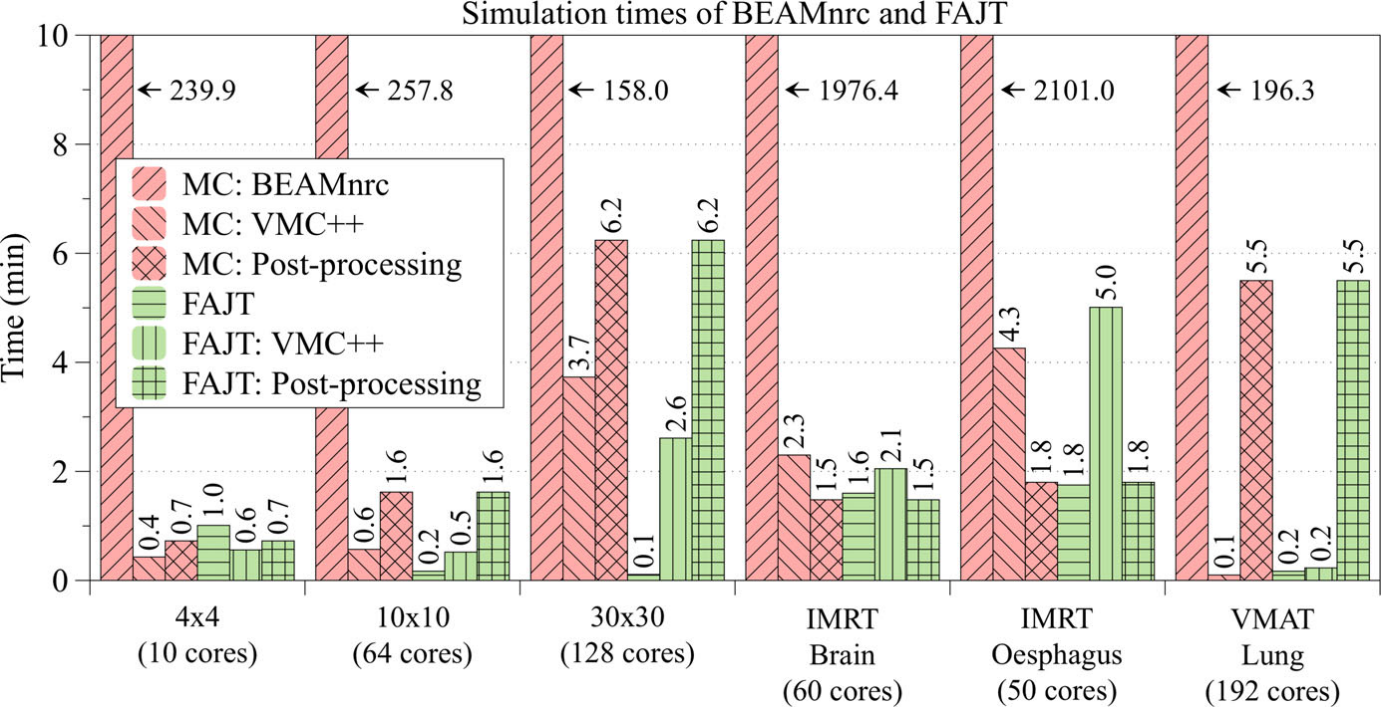
A breakdown of the simulation times for the FAJT method, compared to BEAMnrc. There are three components: modeling of the secondary collimator, dose calculation, and post processing (primarily dose summation). The post processing occurs on only one core (the last to finish).

Simulation times are shown in Fig. 6. The first two components, secondary collimator simulation and dose calculation, were determined by averaging the calculation time over all of the CPU cores used for parallelization. The postprocessing component occurs only on the last core to finish. Note that Fig. 6 presents the average simulation times, which means that the total wall clock time was slightly longer. However, variation between parallel simulations is simply due to random fluctuations and not of interest. Simulation speed of the secondary collimators increased with FAJT compared to BEAMnrc by a factor of 237, 1489, and 1395 for 4 × 4, 10 × 10, and 30 × 30 cm^2^ fields, respectively. The speed‐up factors for secondary collimator simulation of IMRT 1, IMRT 2, and VMAT 1 were 1235, 1201, and 1178, respectively. Such considerable speed‐ups motivate the clinical use of FAJT MC.

## 4 DISCUSSION

This study presents, to the best of our knowledge, the first integration of a flat‐absorbing secondary collimator model with a fast MLC model to enable efficient dose calculations for VMAT and IMRT in jaw‐tracking mode. The short MC dose calculation times have allowed for an integration of this model into a clinical process as a second‐check of VMAT and IMRT plans. Since implementation in August 2015 at our center, our MC software framework with the FAJT model has been used for VMAT/IMRT dose verification of over 1000 treatment plans. Results demonstrated in this paper are shown for a 6 MV beam, but the model has been tested and is in clinical use for all beam energies available in our department: 6 MV, 10 MV‐FFF, 10 MV, and 15 MV. Our clinical computational system utilizes 24‐core servers, different from the resources used for this research, but the calculation times we see on that system are in the same range of 2–10 min as obtained during the performance tests reported in this study for a 64‐core server. These timelines allow for the completion of a second check for a given IMRT/VMAT plan within 20–30 min, which has been found very acceptable. Efficiency of the system could improve further by parallelizing postsimulation summation of the dose distributions.

The presented benchmarks of the FAJT method demonstrated good agreement with BEAMnrc calculations as well as with the portal image‐based dosimetry of EPIdose.^33^ As with most dosimetry methods, the portal image‐based method has its strengths and deficiencies. An advantage of this method is that it captures measured particle fluence that can be processed and used for the dose reconstruction. Therefore, it has valuable information on fluence modulation and MLC transmission, that is often less accurate in computational models. On the other hand, the dose reconstruction employs a relatively simple convolution‐based algorithm that is very accurate in homogeneous media near the beam axis, but suffers reduced accuracy off‐axis due to an invariable convolution kernel used in the process. This is where we see increased differences between EPIDose‐based reconstructed dose and our FAJT MC as well as AAA calculations. We therefore attribute these differences more to the imperfection of EPID and convolution‐based dose reconstruction method than to the inaccuracy of the FAJT model.

Previously, the impact of nine levels of simplification of particle transport through beam collimation systems (jaws and MLCs) was investigated.^21^ The most rigorous of the nine methods was faithful MC simulation using EGSnrc, while the simplest was the “Flat‐Absorbing” method, similar to the FAJT model, but implemented with static jaws and a simpler MLC model. The Flat‐Absorbing method also collimated particles at the vertical midpoint of the jaws, rather than the top surface as in FAJT. In our simulations, this difference did not have statistically significant impact. The authors did not demonstrate dose profiles, but the *γ*‐index agreement for open 10 × 10 cm^2^ fields was similar to the results for the FAJT model. In their implementation, simulation times from the Flat‐Absorbing method were faster than BEAMnrc by a factor of 274 for a 10 × 10 cm^2^ 6 MV field, compared to a factor of 1489 in our implementation. The higher efficiency in our case may be due to storing the phase‐space in RAM, or different algorithm design. However, they chose to use a rather high photon cut‐off in BEAMnrc of PCUT = 0.1 MeV, rather than the PCUT = 0.01 MeV in our simulations. This would significantly reduce the observed speed‐up. The authors used the same Flat‐Absorbing style model to transport radiation through both jaws and MLCs, and the calculated IMRT cases using a Flat‐Absorbing model for jaws and MLC produced relatively low agreement with BEAMnrc: the mean gamma‐index pass rate (1%/1 mm above 1%) over 10 IMRT cases was 67.7%. Note that this result is not directly comparable to this study, due to more stringent criteria and unknown IMRT conditions. The authors commented that it was conceivable to combine different transport methods for jaws and MLCs, and the present work demonstrates one of these cases developed for the purpose of dose verification in the clinical workflow.

Our results show that combination of simple jaw model such as FAJT with fast but accurate MLC model indeed provides very efficient dose calculation option for verification of VMAT and IMRT plans.

## 5 CONCLUSION

The FAJT model was shown to provide a substantial reduction in simulation times at the cost of a small accuracy sacrifice. So long as the user is aware of the accuracy limitations, our implementation of the FAJT secondary collimator model is a valuable addition to the clinical toolset. The FAJT model is suitable for an array of clinical MC dose calculations, particularly treatment plan dose verification where fast calculation times are essential.

## CONFLICT OF INTEREST

The authors have no relevant conflicts of interest to disclose.
